# School Contextual Features of Social Disorder and Mental Health Complaints—A Multilevel Analysis of Swedish Sixth-Grade Students

**DOI:** 10.3390/ijerph15010156

**Published:** 2018-01-19

**Authors:** Bitte Modin, Stephanie Plenty, Sara B. Låftman, Malin Bergström, Marie Berlin, Per A. Gustafsson, Anders Hjern

**Affiliations:** 1 Centre for Health Equity Studies (CHESS), Stockholm University/Karolinska Institutet, SE-106 91 Stockholm, Sweden; sara.brolin.laftman@chess.su.se (S.B.L.); malin.bergstrom@ki.se (M.B.); anders.hjern@chess.su.se (A.H.); 2 Institute for Futures Studies (IFFS), SE-111 31 Stockholm, Sweden; stephanie.plenty@iffs.se; 3 Swedish Institute for Social Research, SE-106 91 Stockholm, Sweden; 4 National Board of Health and Welfare, SE-106 30 Stockholm, Sweden; marie.berlin@socarb.su.se; 5 Department of Sociology, Stockholm University, SE-106 91 Stockholm, Sweden; 6 Department of Clinical and Experimental Medicine (IKE) and Center for Social and Affective Neuroscience (CSAN), Linköping University, SE-581 83 Linköping, Sweden; per.a.gustafsson@liu.se

**Keywords:** social disorganization theory, emotional, complaints, psychosomatic complaints, bullying, victimization, behavioural problems, school context, school climate, multilevel

## Abstract

This study addressed school-contextual features of social disorder in relation to sixth-grade students’ experiences of bullying victimization and mental health complaints. It investigated, firstly, whether the school’s concentrations of behavioural problems were associated with individual students’ likelihood of being bullied, and secondly, whether the school’s concentrations of behavioural problems and bullying victimization predicted students’ emotional and psychosomatic health complaints. The data were derived from the Swedish National Survey of Mental Health among Children and Young People, carried out among sixth-grade students (approximately 12–13 years old) in Sweden in 2009. The analyses were based on information from 59,510 students distributed across 1999 schools. The statistical method used was multilevel modelling. While students’ own behavioural problems were associated with an elevated risk of being bullied, attending a school with a higher concentration of students with behavioural problems also increased the likelihood of being bullied. Attending a school with higher levels of bullying victimization and behavioural problems predicted more emotional and psychosomatic complaints, even when adjusting for their individual level analogues. The findings indicate that school-level features of social disorder influence bullying victimization and mental health complaints among students.

## 1. Introduction

Mental health problems often begin to surface during adolescence and the school environment can play an important role in supporting young people’s well-being. Peer relations and the behaviour of others are stressors that many young people may face difficulties with at some stage. However, some youth experience additional stressors related to the broader social environment at school that can also negatively influence their mental health. The current study investigates the role of behavioural problems in terms of conduct disorder and hyperactivity and bullying victimization as school contextual stressors and how these relate to adolescents’ emotional and psychosomatic health complaints.

Stemming from criminological literature, social disorganization theory [1] proposes that indicators of social disorder in public spaces constitute contextual stressors that can promote fear of crime and result in poorer well-being. Within the neighbourhood context, such indicators include a higher concentration of poverty, vandalism, violence, or residential mobility. For example, witnessing neighbourhood violence has been associated with greater internalising problems, such as depression and anxiety in young residents [2,3].

When applied to the school context, expressions of social disorganization may signal to students that the school social environment is volatile and unsupportive [4,5,6]. Investigations of school disorganization have typically focused on demographic or structural factors, such as school SES, size, distribution of ethnicity and teacher-student ratio. Studies have found that social disorganization captured by such school characteristics appear to undermine the stability of a school and its capacity to promote well-being, as demonstrated by higher bullying rates [4,7] and poorer mental health among its students [7,8].

Comparatively little research has examined the effects of behavioural measures of school disorganization on student health. School climate research shows that perceiving a supportive social environment is associated with less bullying victimization [9,10] and better self-rated mental health among students [11,12,13]. A positive school climate reflects social dynamics (often between students and teachers) and norms that are not experienced as threatening. However, the specific behaviours that contribute to social disorganization need closer examination. A poor disciplinary environment in terms of unclear rules and a lack of following and enforcement of school rules can promote a culture of bullying [5,10,14,15]. As persistent exposure to others’ bullying or disruptive behaviour is likely to communicate to students that antisocial behaviours are tolerated, concerns about one’s own vulnerability may be heightened [16]. Indeed, research indicates that observing bullying among classmates is associated with poorer mental health, even among those who are not bullied themselves [13,17,18,19,20]. While psychosocial adjustment problems have been recognized as a risk factor for being bullied [21,22], behavioural problems involving destructive, defiant, aggressive or hyperactive conduct have also been identified as key risk factors for future poorer mental health [23], potentially through strained peer relations. In line with this, there is growing evidence that young people who are bullied are more likely to exhibit behavioural problems than other young people [24,25,26,27].

However, it is important to consider both individual and school-level effects in these associations because they reflect different types of social processes. Through social disorganization processes, the presence of bullying and other behavioural problems in a school (i.e., school contextual social disorder) may create a stressful social environment and elevate students’ anxiety, thus “spilling over” to other students’ well-being. Therefore, greater bullying and behavioural problems in a school may be associated with poorer mental health among students irrespective of their own behavioural problems and experiences of bullying victimization.

Accordingly, focussing on school-aggregated measures of behavioural problems and bullying victimization whilst simultaneously adjusting for their student-level analogues as well as the sociodemographic composition of schools, the following research questions will be addressed:

(1) Is a larger concentration of students with behavioural problems within a school associated with an increased risk of bullying victimization among its students?
(2) Is a larger concentration of students with behavioural problems within a school associated with more emotional and psychosomatic health complaints among its students?
(3) Is a larger concentration of students who are bullied within a school associated with more emotional and psychosomatic health complaints among its students?

## 2. Methods

### 2.1. Participants and Procedure

The study draws on data from the Swedish National Survey of Mental Health among Children and Young People carried out in 2009 [28,29]. The survey covered 80,671 students in grade 6 (2853 schools), corresponding to 83% of the grade 6 students enrolled that year. Special needs schools, hospital schools and other special education units were not included. For the current analyses, 21,161 students (26.2%) were excluded due to questionnaire incompletion (18.4%), missing information about school’s sociodemographic characteristics (5.1%) as well as poor school-sample representation (2.7%), i.e., schools with fewer than 10 participating students were removed from analysis. This resulted in an analytical sample of 59,510 students distributed across 1999 schools, reflecting 61.2% of all grade 6 students registered in Sweden during 2009.

### 2.2. Measures

The current study focused on behavioural problems and bullying victimization, as well as two types of mental health complaints: emotional and psychosomatic complaints.

#### 2.2.1. Individual-Level

Behavioural problems were examined using the conduct and hyperactivity subscales of the Strengths and Difficulties Questionnaire (SDQ) [30]. The following five items addressed conduct problems: ‘I get very angry and often lose my temper’, ‘I usually do as I am told’, ‘I fight a lot’, ‘I can make others do what I want’, ‘I am often accused of lying or cheating’ and ‘I take things that are not mine, e.g., from school or elsewhere’. The following five items addressed hyperactivity: ‘I am restless, I cannot stay still for long’, ‘I am constantly fidgeting or squirming’, ‘I am easily distracted, I find it difficult to concentrate’, ‘I think before I do things’, ‘I finish the work I’m doing. My attention is good.’ The three response options were ‘Not true’ (1), ‘Somewhat true’ (2) and ‘Certainly true’ (3). An index of behavioural problems ranging from 10 to 30 was generated by summing responses to all items (Cronbach’s alpha = 0.69, mean = 15.1, s.d. = 3.0).

Bullying victimization was based on a three items subscale (social acceptance and bullying) from the KIDSCREEN-52 quality of life questionnaire [31,32]: If you think about last week … ‘Have you been afraid of other students?’, ‘Have other students made fun of you?’, and ‘Have other students bullied you?’ The response options included ‘Never’, ‘Seldom’, ‘Quite often’, ‘Very often’ and ‘Always’. Responses were first summed to reflect a score ranging from 3 to 15, then dichotomized to reflect students who were bullied (scores 8–15) versus those who were not bullied (scores 3–7). 

Emotional complaints were assessed using 13 items also from the KIDSCREEN-52 instrument [32,33]. The following six items were drawn from the emotional well-being subscale: If you think of last week … ‘Has your life been enjoyable?’, ‘Have you felt pleased that you are alive?’, ‘Have you felt satisfied with your life?’, ‘Have you been in a good mood?’, ‘Have you felt cheerful?’ and ‘Have you had fun?’ The five response options to the first three items were: ‘Not at all’, ‘Slightly’, ‘Moderately’ ‘Very’, and ‘Extremely’, while response options for the latter four items were: ‘Never’, ‘Seldom’, ‘Quite often’, ‘Very often’, and ‘Always’. The following seven items come from the moods and emotions subscale: If you think of last week … ‘Have you felt that you do everything badly?’, ‘Have you felt sad?’, ‘Have you felt so bad that you didn’t want to do anything?’, ‘Have you felt that everything in your life goes wrong?’, ‘Have you felt fed up?’, ‘Have you felt lonely?’ and ‘Have you felt under pressure?’ The response options included ‘Never’, ‘Seldom’, ‘Quite often’, ‘Very often’, and ‘Always’. Responses were summed to create an emotional complaints index ranging from 13 to 65 (Cronbach’s alpha = 0.93, mean = 21.7, s.d. = 7.8).

Psychosomatic complaints were captured by the PsychoSomatic Problems scale (PSP) [33], which is based on eight items in its original version. We excluded one item (difficulty in concentrating) due to overlap with an item in the predictor variable (behavioural problems). The following seven questions were used: If you think about the last 6 months … ‘Have you felt that you had trouble sleeping?’, ‘Have you been bothered by headaches?’, ‘Have you been bothered by stomach pain?’, ‘Did you feel tense?’ ‘Have you lacked appetite?’, ‘Have you felt sad?’ and ‘Have you felt dizzy?’ The response categories were ‘Never’, ‘Seldom’, ‘Quite often’, ‘Very often’, and ‘Always’. A summed score was formed ranging from 7 to 35 (Cronbach’s alpha = 0.85, mean = 13.4, s.d. = 5.1). 

#### 2.2.2. School-Level

Within each school, the mean response on bullying victimization and behavioural problems were calculated to form aggregated school-level measures.

As proxy controls for school sociodemographic factors are often associated with student mental health, school-specific information about the proportion of students who were born abroad and the proportion of parents with a post-secondary education was included in the analyses. This information was retrieved from The Swedish National Agency for Education’s official database [34].

### 2.3. Data Analysis

In the analyses where bullying victimization serves as the dependent variable, two-level binary logistic regression models were performed using the ‘xtmelogit’ command in Stata 14. First, the between-school variation in bullying victimization was calculated through an empty model. Two additional models were fitted. In Model 1, we examined if behavioural problems at the individual level increased the risk of being bullied. In Model 2, the aggregated school-level measure of behavioural problems was added in order to assess whether there were any additional school contextual effects of behavioural problems on bullying victimization. In Model 2, also the school-level control variables measuring school sociodemographic characteristics were included. In all models, the intra class correlation coefficient (ICC) was calculated. ICC for binary outcomes provides approximate information on the share of the total variation in the outcome variable that can be attributed to the school-level.

Emotional and psychosomatic complaints were analysed by means of two-level linear regression models, using the ‘xtmixed’ command in Stata 14. Scores for emotional and psychosomatic complaints were transformed into z-scores (mean value = 0, standard deviation = 1) so that results could be expressed in standard deviations and more easily comparable across the two outcomes. Also for these outcomes, an empty model was first fitted in order to evaluate the between-school variation in emotional and in psychosomatic complaints. Four additional models were estimated for each outcome. Model(s) 1 included gender and bullying victimization at the individual level. Model(s) 2 added behavioural problems at the individual level. Model(s) 3 added the aggregated school-level measures of bullying victimization and of behavioural problems, and Model(s) 4 finally controlled for school-level sociodemographic characteristics. ICC was calculated for all models, presenting the proportion of variation in the dependent variable that can be attributed to school-level differences.

## 3. Results

Descriptive statistics of the sample are presented in Table 1. Both emotional and psychosomatic complaints were positively skewed with the majority of students reporting few complaints. Overall, 4.8% of the students were classified as bullied, while the school-level mean of bullying varied between 3.0 and 6.3. The mean score for behavioural problems was 15.1 at the individual level, and ranged between 12.5 and 18.8 across schools. The control variables reflected a fairly wide distribution in sociodemographic characteristics across schools, with the proportion of foreign-born students ranging between 0 and 58% and the proportion of highly educated parents ranging between 16 and 94%.

Table 2 presents estimates from the multilevel binary logistic regression analyses predicting bullying victimization. The empty model shows that there were statistically significant between-school differences in bullying victimization with an ICC corresponding to 5.5%. Model 1 demonstrates that girls had significantly higher odds (30%) of being bullied than boys when behavioural problems were adjusted for. Students’ own behavioural problems significantly increased the likelihood of being bullied, with one standard deviation increase in the behavioural problems index corresponding to a more than doubled odds of being bullied. Model 2 demonstrates that school-level behavioural problems were positively and significantly associated with an increased likelihood of being bullied, even when controlling for students’ own behavioural problems. Model 2 also shows that the effects of behavioural problems at both the student- and the school-level were statistically significant even when controlling for school sociodemographic characteristics. Larger school proportions of foreign background students were associated with an increased likelihood of bullying and larger school proportions of highly educated parents were linked with a reduced likelihood of bullying.

Table 3 displays results from the multilevel linear regressions predicting emotional and psychosomatic complaints. The two empty models show that a significant proportion of variance in emotional complaints (2.9%) and in psychosomatic complaints (1.8%) was due to differences between schools. As shown in Models 1a–1b, being a girl and being bullied were associated with more emotional and psychosomatic complaints. Greater behavioural problems at the individual level predicted more emotional and psychosomatic complaints, even when adjusting for bullying victimization (Models 2a–2b). Furthermore, for both outcomes, the estimates for bullying victimization were reduced when behavioural problems was added to the models, although they both remained strong and statistically significant. Models 3a–3b added the aggregated school-level measures of bullying victimization and of behavioural problems. Larger school means of bullied students predicted greater emotional and psychosomatic complaints, as did higher concentrations of behavioural problems. As shown in Models 4a–4b, the school-level estimates of bullying victimization and of behavioural problems remained robust and statistically significant even when adjusting for school sociodemographic factors. The control variables demonstrated that attending a school with a larger proportion of foreign-born students was associated with fewer emotional and psychosomatic complaints, while attending a school with a larger share of students with highly educated parents was associated with more emotional and psychosomatic complaints.

## 4. Discussion

This study examined features of school-contextual social disorder in relation to sixth-grade students’ experiences of bullying victimization and mental health complaints, both of which are important precursors of later expressions of severe mental ill health [35,36] and their related risk behaviours [37,38,39]. In line with social disorganization theory [1], the findings indicate that a school environment characterized by social disorder undermine students’ emotional and psychosomatic well-being. 

With regards to bullying victimization, we found that at the individual level, behavioural problems increased the risk of being bullied, reflecting findings from earlier studies [24,25,26,27]. At the school-level, greater social disorder measured as the school concentration of behavioural problems was associated with a greater likelihood of bullying victimization, regardless of students’ own behavioural problems. An interpretation of this finding is that a social climate characterized by poor conduct may have less cohesiveness between students, which may inadvertently promote a culture with a greater tolerance of bullying [40]. It is possible that students in schools characterized by social disorder are more likely to have normative beliefs that bullying is acceptable, which has been shown to be positively linked with bullying behaviour [9]. The interpretation is also consistent with previous studies reporting that students who perceive clear and consistent school rules tend to report feeling safer [41] and less exposed to bullying victimization [14,15,20].

The analyses also showed that sociodemographic characteristics of the school were linked with both bullying victimization and mental health complaints. In accordance with previous research on Swedish data [42,43], the school-level proportion of students born abroad was positively associated with bullying victimization, but negatively associated with mental health. Conversely, the proportion of students with parental post-secondary education was negatively linked with bullying victimization, but positively associated with mental health complaints. However, the latter association emerged only when bullying victimization and behaviour problems were included in the model. Thus, the crude estimate for school-level parental education demonstrated a weak negative association with student health (data not shown).

The study also demonstrated clear links between social disorganization at the school-level and students’ mental health complaints. Larger concentrations of behavioural problems and of bullying victimization were associated with more emotional and psychosomatic complaints, even when adjusting for students’ own behavioural problems and experiences of bullying victimization. These results reflect and extend those from earlier studies which showed that the occurrence of bullying victimization among classmates is associated with poorer mental well-being, even among students who are not directly exposed themselves [17,18,19,20]. A possible interpretation of these findings is that the presence of bullying and other behavioural problems in a school is a stressor for the student body as a whole, and as such may lead to an overall increase of emotional and psychosomatic problems in the student population [44]. 

This study was based on a total sample of Swedish sixth-graders, and thus the findings present a picture of the broader student population that is rarely captured. Nevertheless, there are also limitations. The data were cross-sectional and investigations of longitudinal associations are needed to better understand causal links and long-term impacts of social stressors on student well-being. Although KIDSCREEN-52’s social acceptance and bullying subscale is a well-established and commonly applied measure of bullying victimization [32,42,45], the use of a continuous variable to assess whether a student is bullied or not was not considered optimal. Therefore, we dichotomized the index using a cut-off value of ≥8 in order to capture only a small group of students. Nearly 5% of the students were classified as being bullied according to this operationalization, which is very close to the proportion of 13-year-old students who reported being bullied at least two or three times a month in the Swedish version of Health Behaviors in School-aged Children in 2009/2010 and 2013/2014 [46]. Our study addressed bullying in general, rather than specific types of bullying (such as physical, relational or cyber). Future research should also examine the implications that different types of bullying within a school may have for student health. For example, although cyberbullying occurs outside the immediate school context, having a large prevalence of students experiencing this type of bullying may still translate to poorer social organization within a school. It would also be beneficial to examine the effects of problem behaviours for both victims and bully-victims. Although we found that students’ own behavioural problems predicted bullying victimization, previous research has found that aggressive behaviours are more characteristic of bully-victims than of victims [27]. The current data did not have information on bullying perpetration and so differences in ‘victim status’ was not possible to examine.

Finally, in an international perspective, Sweden has very low rates of self-reported bullying victimization compared to other countries [47,48] and therefore cross-cultural generalizability of the findings may be somewhat limited. It may be the case that the contextual effects of bullying victimization are even greater in countries where bullying is more common. Future research could address cultural differences in social stressor effects on student’s mental health.

## 5. Conclusions

This study applied social disorganization theory to the school context by focusing on behaviour problems in relation to sixth-grade students’ experiences of bullying victimization and mental health complaints. It demonstrated that a school context characterized by social disorder is associated with an increased risk of bullying victimization and with more mental health complaints, irrespective of the students’ own behavioural problems and experiences of bullying victimization as well as school sociodemographic factors.

The findings are consistent with the assumptions of social disorganization theory [1] in that contextual stressors were shown to be associated with poorer individual well-being. They underline the importance of considering how the broader social climate in school may function adversely or favourably for students’ well-being. School-contextual social characteristics, such as behavioural problems and bullying, present factors that are potentially modifiable through policy or practice changes. Important prerequisites for promoting a prosperous social school environment include a strong school leadership [49], a strong school ethos [50], as well as clarity of school rules and clear disapproval of bullying [10]. We conclude that promoting a social climate characterized by condemnation of bullying and by efficient handling of disruptive behaviours would benefit the well-being of all students.

## Figures and Tables

**Table 1:** Descriptive statistics for student- and school-level variables.

Variables	Range	Mean or %
Student-Level		
Gender: girls	0, 1	50, 6%
Bullying victimization	0, 1	4, 8%
Behavioural problems (z-score)	−1.7, 4.9	0 (1)
Emotional complaints (z-score)	−1.1, 5.5	0 (1)
Psychosomatic complaints (z-score)	−1.3, 4.2	0 (1)
School-Level		
Bullying victimization (mean)	3.0, 6.3	3.9 (0.4)
Behavioural problems (mean)	12.3, 18.8	15.1 (0.7)
Students born abroad (%)	0, 58	7.3 (7.0)
Parents with post-secondary education (%)	16, 94	51.1 (15.8)
*N* (students)	59,510	
Internal attrition (students)	21,161	
*N* (schools)	1999	
Internal attrition (schools)	857	

**Table 2:** Multilevel logistic regressions predicting bullying victimization (59,510 sixth-grade students distributed across 1999 schools).

Variables	Odds Ratios		
	Empty Model	Model 1	Model 2
Student-Level			
Girls (versus boys)		1.30 ***	1.30 ***
Behavioural problems (standardized)		2.08 ***	2.04 ***
School-Level			
Behavioural problems (mean)			1.11 ***
Students born abroad (%)			1.02 ***
Parents with post-secondary education (%)			0.99 ***
Between-school variance	0.193 ***	0.145 ***	0.087 ***
Intraclass correlation coefficient (ICC)	5.5%	4.2%	2.6%

*** *p* < 0.001.

**Table 3:** Multilevel linear regressions predicting emotional and psychosomatic complaints (59,510 sixth-grade students distributed across 1999 schools).

Variables	Emotional Complaints (*β)*					Psychosomatic Complaints (*β)*				
	Empty	Model 1a	Model 2a	Model 3a	Model 4a	Empty	Model 1b	Model 2b	Model 3b	Model 4b
Student-Level										
Girls (versus boys)		0.27 ***	0.35 ***	0.35 ***	0.35 ***		0.32 ***	0.41 ***	0.41 ***	0.41 ***
Bullying victimization		1.44 ***	1.13 ***	1.12 ***	1.12 ***		1.17 ***	0.85 ***	0.84 ***	0.84 ***
Behavioural problems			0.39 ***	0.38 ***	0.38 ***			0.40 ***	0.40 ***	0.40 ***
School-Level										
Bullying victimization (mean)				0.05 ***	0.08 ***				0.06 ***	0.06 ***
Behavioural problems (mean)				0.04 ***	0.04 ***				0.02 **	0.02 *
Students born abroad (%)					-0.003 **					-0.001 *
Parents with post-secondary education (%)					0.002 ***					0.001 ***
Between-school variance	0.029 ***	0.026 ***	0.016 ***	0.015 ***	0.013 ***	0.018 ***	0.017 ***	0.011 ***	0.010 ***	0.009 ***
Intraclass correlation coefficient (ICC)	2.9%	3.0%	2.3%	2.1%	1.7%	1.8%	1.8%	1.5%	1.4%	1.3%

*** *p* < 0.001, ** *p* < 0.01, * *p* < 0.05.
